# Chilean Gastric Cancer Task Force: A study protocol to obtain a clinical and molecular classification of a cohort of gastric cancer patients

**DOI:** 10.1097/MD.0000000000010419

**Published:** 2018-04-20

**Authors:** Gareth I. Owen, Mauricio P. Pinto, Ignacio N. Retamal, María F. Fernádez, Betzabe Cisternas, Sebastian Mondaca, Cesar Sanchez, Hector Galindo, Bruno Nervi, Carolina Ibañez, Francisco Acevedo, Jorge Madrid, José Peña, Maria Loreto Bravo, Maria Jose Maturana, Miguel Cordova-Delgado, Diego Romero, Nathaly de la Jara, Javiera Torres, Maria Rodriguez-Fernandez, Manuel Espinoza, Carlos Balmaceda, Matías Freire, Valentina Gárate-Calderón, Fernando Crovari, Paula Jimenez-Fonseca, Alberto Carmona-Bayonas, Ariel Zwenger, Ricardo Armisen, Alejandro H. Corvalan, Marcelo Garrido

**Affiliations:** a Hematology and Oncology Department, Faculty of Medicine, Pontificia Universidad Católica de Chile (PUC); b Department of Physiology, Faculty of Biological Sciences, PUC; c Biomedical Research Consortium of Chile; d Millennium Institute on Immunology and Immunotherapy, PUC; e Core Biodata, Advanced Center for Chronic Diseases, PUC; f Center UC Investigation in Oncology, PUC; g Department of Pathology, Faculty of Medicine, PUC; h The Institute for Biological and Medical Engineering, PUC; i Center of Clinical Research, Health Technology Assessment Unit, PUC; j Department of Public Health, PUC; k Center of Excellence in Precision Medicine, Macul; l Department of Gastrointestinal Surgery, PUC, Santiago, Chile; m Hospital Universitario Central de Asturias, Oviedo; n Hospital Morales Meseguer, Murcia, Spain; o Hospital Provincial de Neuquén, Neuquén, Argentina.

**Keywords:** cancer subtypes, chemotherapy, gastric adenocarcinoma, gastric cancer, immunotherapy, molecular classification, prognosis, survival

## Abstract

**Abstract:**

Gastric cancer (GC) is the world's second-leading cause of neoplastic mortality. Genetic alterations, response to treatments, and mortality rates are highly heterogeneous across different regions. Within Latin America, GC is the leading cause of cancer death in Chile, affecting 17.6 per 100,000 people and causing >3000 deaths/y. Clinical outcomes and response to “one size fits all” therapies are highly heterogeneous and thus a better stratification of patients may aid cancer treatment and response.

The Gastric Cancer Task Force is a Chilean collaborative, noninterventional study that seeks to stratify gastric adenocarcinomas using clinical outcomes and genomic, epigenomic, and protein alterations in a cohort of 200 patients. Tumor samples from the Pathology Department and the Cancer Center at UC-Christus healthcare network, Pontificia Universidad Católica de Chile will be analyzed using a panel of 143 known cancer genes (Oncomine Comprehensive Assay) at the Center of Excellence in Precision Medicine in Santiago, Chile. In addition, promoter methylation for selected genes will be performed along with tissue microarray for clinically relevant proteins (e.g., PD-L1, Erb-2, VEGFR2, among others) and *Helicobacter pylori* and Epstein–Barr virus status. Obtained data will be correlated to 120 clinical parameters retrieve from medical records, including general patient information, cancer history, laboratory studies, comorbidity index, chemotherapy, targeted therapies, efficacy, and follow-up.

The development of a clinically meaningful classification that encompasses comprehensive clinical and molecular parameters may improve patient treatment, predict clinical outcomes, aid patient selection/stratification for clinical trials and may offer insights into future preventive and/or therapeutic strategies in patients from Latin America region.

**Trial registration:**

ClinicalTrials.gov Identifier: NCT03158571, Registered on May 18, 2017.

## Abstract 2

Supplemental Digital Content is available in the text

## 1 Background

**Figure 1:**
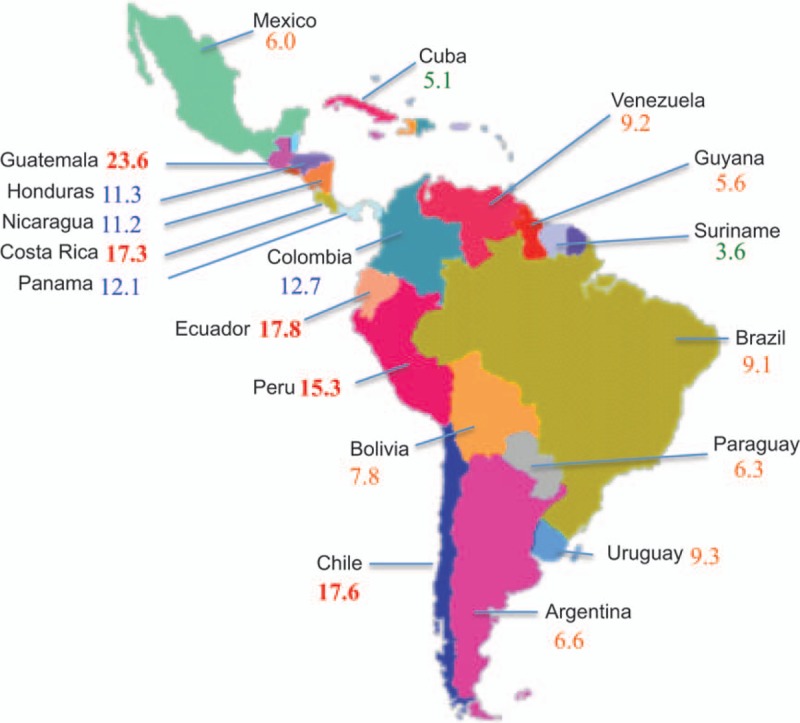
Stomach cancer age adjusted death rates per 100,000 habitants/y in Latin America. Source: WHO, 2014. http://www.worldlifeexpectancy.com/cause-of-death/stomach-cancer/by-country/.

Stomach or gastric cancer (GC) is the world's second-leading cause of cancer death.^[1–3]^ It is characterized by a regional and geographical heterogeneity: in regions such as North America and Western Europe, the number of deaths has steadily decline in recent decades, in contrast in many Asian and Latin American countries, mortality rates have remained high.^[2,4]^ It has been consistently shown that socioeconomic status is closely linked to GC incidence.^[5]^ In recent years, Chile has been one of the Latin American countries with faster economic growth. Consequently, GC mortality rates have fallen, but not to the extent of other countries in a similar transition. In fact, GC is still the country's leading cause of cancer death with 17.6 cases per 100,000 habitants/y^[6]^ (Fig. 1). In most cases, this is explained by late stage diagnoses.^[7]^ Indeed, >50% of patients are diagnosed at an advance stage of the disease.^[8]^ Given Chile's long coastline (and thus varying terrains) and the presence of an indigenous population,^[9]^ there are several aspects relating to GC incidence and mortality that cannot be extrapolated from studies carried out in other populations.

As pointed out, GC mortality rates are highly heterogeneous across different geographical regions with higher mortality rates in Latin American and Asian countries located along the Pacific Rim including Chile, South Korea, and Japan, with the exception of the North American countries such as the United States and Canada. The heterogeneity of GC mortality rates across Latin America is illustrated in Fig. 1, in which rates range from 3.6 to 23.6 per 100,000 people. In the region, Chile ranks third with a mortality of 17.8/100,000 inhabitants/y. This asymmetrical distribution of GC mortality rates therefore calls for a specific assessment on a country-by-country basis.^[4]^

GCs comprise tumors located beneath the gastroesophageal junction. In general, these tumors are classified as gastric adenocarcinomas (GACs) and are characterized by an asymptomatic slow-progression.^[10]^ Currently, available GAC treatments include surgery, chemo, radio, and immunotherapy, either alone or in combination. Potentially effective curative strategies involve partial or total gastrectomy,^[1]^ followed by lymphadenectomy.^[11]^ Despite this, 40% to 65% of patients exhibit recurrent disease and thus 5-year overall survival (OS) considering all stages only ranges between 10% and 30%.^[10]^ In the case of metastatic disease, median OS rarely exceeds 12 months in most international series.^[1]^

Given the success of preventive care strategies implemented in other countries such as Japan,^[12]^ the Ministry of Health for Chile strongly advocates for GAC prevention and early detection as a national priority in public health policies.^[13]^ However, the continued poor survival indicators, along with its high incidence in Chile, suggest that it is imperative to undertake a new course of action and to adopt novel, more appropriate measures to confront this complex, multifactorial and devastating disease. While current efforts to reduce mortality rates should focus on disease prevention and early diagnosis, these health measures are expected to take years to make a significant impact, thus it can be assumed that the incidence of GC in Chile will remain high into the next decade. While priorities should focus on prevention and early stage diagnosis, efforts should also be placed on the optimization of therapies available for potentially curable tumors. This implies the need to develop new methods of classification that allows the prediction of clinical results, and thus a more optimal selection of treatments. Hence, both Chile and the cancer community require a stratification of patients based centrally around clinical outcomes (such complementary therapies and postsurgery chemotherapy). This, in accordance with relative risk factors, genetics and epigenetics may aid the selection of the most appropriate drugs according to tumor type or potential targets.^[14]^

Currently, patients undergoing chemotherapy regimens are selected based on: recurrence risk rate (depending on lymph node or stomach wall infiltration), patient type (age, toxicity risk, and comorbidities), and tumor histological type or molecular markers (Lauren's diffuse or HER2 expression); using this methodology 35% of stage II/III patients achieve 5-year survival,^[15]^ and 18% of stage IV patients reach 2-year survival.^[16]^ Two important conclusions are derived from these data: first, efforts must be made to optimize these results and second, an efficient patient selection may improve these numbers.

Histopathologically, GACs can be divided into diffuse and intestinal subtypes.^[17]^ Although diffuse tumors have a worse prognosis and a different pattern of dissemination, with the possible exception of the lower benefit of adjuvant radiotherapy, it is unclear how histology can influence the treatment decision. The World Health Organization has proposed an alternative system and subdivides GACs as papillary, tubular, mucinous, and poorly cohesive carcinoma.^[18]^ However, in many cases, similar histological types display disparate treatment response rates and prognoses.

Consequently, current classification systems have little or no relevance in terms of clinical management of the patient and therefore the development of a clinically meaningful classification that will allow more effective treatments for patients is urgently needed.

There exists a long-established association between GC and infectious agents such as the bacterium *Helicobacter pylori*^[19,20]^ and the Epstein–Barr virus (EBV).^[21]^ Both the histological distribution of cancer subtypes and the frequency of *H pylori* and EBV are variable throughout the world.^[22,23]^ In Chile, *H pylori* is carried by the majority of the population,^[24]^ while EBV is associated with approximately 16% of GAC cases^[25]^ which is higher than that of most other countries, both regionally and worldwide.^[24–27]^ These statistics in themselves warrant a closer examination of the molecular variants and clinical profiles of EBV-associated GC cases in Chile.

Interestingly, 3 major studies have used large cohorts of patients and have established molecular GC subtypes using tumor samples and/or cell lines: Studies by the Duke-National University of Singapore,^[28]^ the Cancer Genome Atlas (TCGA) study,^[29]^ and the Asian Cancer Research Group (ACRG)^[30]^ have profiled 37 GC cell lines (validated in 521 patients), or 295 and 300 patients, respectively.

In particular, the ACRG study defines 4 subtypes: microsatellite instability (MSI), microsatellite stable (MSS)/epithelial to mesenchymal transition, MSS/TP53+ and MSS/TP53− tumors, and correlates them with survival rates using 3 different patient cohorts, including TCGA.^[30]^ Other genetic alterations, such as single nucleotide polymorphisms (SNPs) in genes of 2 enzymes, the dihydropyrimidine dehydrogenase (*DPYD*) and the thymidylate synthetase (*TYMS*), can increase the risk of adverse reactions to the 5-fluorouracil (5-FU), a chemotherapy widely used in the treatment of GC both in Chile and worldwide.^[31–33]^

Although all the above-mentioned cohort studies have successfully defined GC subtypes based on expression profile, mutations, genomic rearrangements, and MSI, their correlation with clinical parameters and patient outcomes remain to be fully elucidated. Our project described herein aims to take the first steps into the identification of GAC subgroups in a cohort of 200 Chilean patients. The analysis will profile 143 known cancer genes included on the Oncomine Comprehensive Array (Thermo Fisher, Waltham, MA) and constitutes the first prevalence study on actionable (or “druggable”) targets in Chilean GAC patients.

GCs, like most malignancies are characterized by aberrant expression and/or overexpression of certain proteins and some of these have been used for specific targeted therapies, such as ramucirumab, inhibiting vascular endothelial growth factor receptor-2 (VEGFR2),^[34]^ or trastuzumab targeting the human epidermal growth factor receptor-2 (HER2 or ErbB2).^[35]^ Currently, the confirmed presence of programmed death–ligand 1 (PD-L1) in tumors also predicts better outcome when using checkpoint inhibitor immunotherapies such as pembrolizumab.^[36]^

In summary, the project seeks to correlate genetic alterations, certain SNPs, gene promoter methylation, protein expression levels (by tissue microarray [TMA]), and recorded clinicopathological patient characteristics. This study takes the first steps toward the development of more rational treatment options for Chilean patients, with the promise to offer more meaningful clinical outcomes. Furthermore, information from this study protocol will give the first indication on the percentage of Chilean patients that could receive benefit from targeted therapies and delineate potential future GC prevention strategies.

## 2 Methods

### 2.1 Participating entities

The Chilean Gastric Cancer Task Force (GCTF) is a collective effort between 2 principal entities: The Center of Excellence in Precision Medicine (CEMP), which was established through a joint funding by the government agency for economic development (corporation for the improvement of productivity [CORFO]) and Pfizer Chile and The Center UC for Investigation in Oncology (CITO) based at the Pontificia Universidad Católica de Chile. Both entities are nonprofit research organizations aimed at enhancing public education and implementing strategies to improve clinical outcomes in oncology treatment and cancer prevention.

### 2.2 Primary objective

**Figure 2:**
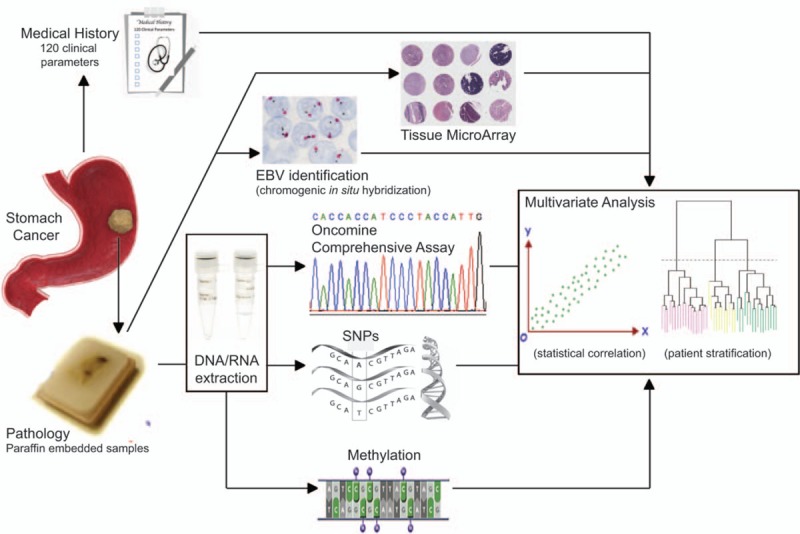
Workflow diagram of the gastric cancer task force. Briefly, GC patient FFPE sections from archived samples are used for TMA analysis and EBV identification. In addition, DNA/RNA is extracted from FFPE samples for NGS, SNPs, and methylation analyses. Genomic and expression profiles of patients are correlated with 120 clinical parameters obtained from patients’ medical records by multivariate analysis. For further details please refer to text. EBV = Epstein–Barr virus, FFPE = formalin-fixed paraffin embedded, GC = gastric cancer, NGS = next generation sequencing, SNP = single nucleotide polymorphism, TMA = tissue microarray.

To stratify GC patients into prognostic subgroups and to correlate therapy response according to clinical, protein, epigenetic, and genetic alterations in a cohort of 200 GAC patients. Figure 2 summarizes the workflow of the GCTF study.

### 2.3 Secondary objectives

- To determine the mutation profile in GC patients.
- To assess the percentage of GC patients that could benefit from currently available “druggable” targets (actionable genes).
- To correlate molecular variants of EBV and clinical profiles in EBV-associated GC cases.
- To assess the expression levels of proteins associated with molecular stratifications and currently targeted therapies (e.g., PD-L1 and antiangiogenics).
- To determine the profile of SNPs in the DPYD and TYMS genes in GC patients and their correlation with adverse events.

### 2.4 Study design

#### 2.4.1 Patient recruitment and characteristics

Diagnosed GC patients will be recruited from the Red UC Christus network in Santiago, Chile. Patient recruitment and signing of informed consent forms, maintenance and monitoring of patient medical records, biological material, and sample extractions will be managed by CITO.

Patient and treatment history reveals that besides surgery and chemotherapy, approximately 10% of patients received trastuzumab (ERBB2 targeted therapy, also called Herceptin), another 10% received immunotherapy including pembrolizumab and ipilimumab (checkpoint inhibitors). Finally, approximately 5% of patients received antiangiogenic therapy (consisting of VEGFR2 targeted therapy with ramucirumab). In addition, histological analysis showed that approximately 50% of patients were classified as intestinal type, 30% as diffuse, and 20% were either mixed or undetermined.

#### 2.4.2 Inclusion criteria

- Adult male or female, aged >18 years.
- Diagnosed with GC (histological or cytological).
- Attending health centers of the Red UC Christus network for at least 3 months with clinical follow-up.
- Capable to read and speak Spanish.
- Willing and able to provide written informed consent to the study that should be dated and signed at the time of enrollment.

#### 2.4.3 Exclusion criteria

Patients:

- With small biopsy samples insufficient for analysis.
- Whose medical records cannot be collected or are unavailable.
- Without signed informed consent.

#### 2.4.4 Clinical data

Clinical data from patients will be obtained by healthcare providers and entered into an online electronic platform at www.clinicaldata.cl. Samples will be coded and patient identity known only to the attending physician. Clinical variables are divided into sections: General Patient Information, Cancer History, Laboratory Studies, Comorbidity (Charlson) Index, Chemotherapy, Efficacy and Follow Up, and Toxicity. A detailed description of General Patient Information, Cancer History, Laboratory Studies, and Comorbidity index including clinical variables and data entered into the platform via questionnaire are shown in Supplementary Table S1. Patient chemotherapy will be classified by: regime, number of cycles, and time of treatment and chemotherapy dose-intensity during the first 6 months. Chemotherapy descriptions are listed in Supplementary Table S2, with chemotherapy regime representing the first-line chemotherapy prescribed to the patients. Complementary data with the number of cycles of chemotherapy and the time of treatment are listed in Supplementary Table S3. Full chemotherapy dose intensity during the first 6 months will be obtained through patient interviews and entered directly into the online platform. Finally, efficacy and follow-up and toxicity data obtained from patients using the questionnaire are listed in Supplementary Tables S4 and S5, respectively.

#### 2.4.5 Main clinical outcomes

Main outcomes will be inferred from obtained clinical data, these include OS, progression-free, and recurrence-free survival rates.

#### 2.4.6 Biological samples and Oncomine comprehensive assay

Biological materials obtained at the Red UC Christus will be transported to CEMP in Santiago de Chile under standardized protocols. A total of >200 patient tumor samples will be obtained from archived formalin-fixed paraffin embedded samples. Nucleic acids will be extracted using the RecoverAll kit (Thermo Fisher, Catalog no. AM1975) and analyzed using the commercially available Oncomine Comprehensive Assay kit. This assay simultaneously analyzes DNA and RNA from samples allowing the assessment of 73 gene hotspots (based on DNA), 49 focal copy number variations (CNVs, DNA based), 26 full coding sequences (for mutations and CNV loss), and 22 gene fusion drivers (RNA). Analyzed genes are summarized in Table 1. Notably, 72 of these genes are drug targets. Genomic raw data obtained (.vcf and .pdf files) will be stored and backed up in a local Data Center for subsequent genomic analysis. Upon publication of the findings of this study, the Oncomine results along with clinical classification of individual tumors will be made publicly available.

**Table 1:**
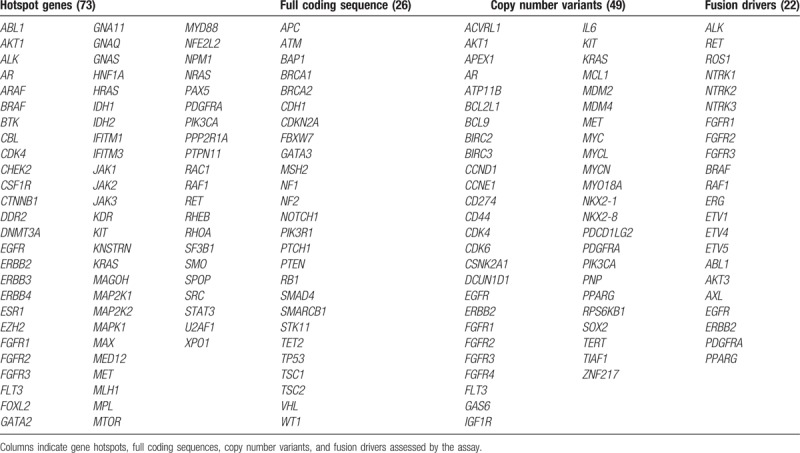
Oncomine comprehensive assay profiled genes by next generation sequencing.

#### 2.4.7 Tissue microarray analysis

The following genes will be further analyzed by a TMA using specific antibodies against: PD-L1 (Dako, Santa Clara CA, Catalog no. SK00521), PD-L2 (Thermo, Catalog no. B7-DC/CD273), Phosphorylated mTOR (Abcam, Cambridge, UK, Catalog no. AB118815), p53 (Catalog no. 5278074001), VEGFR2 (Abcam, Catalog no. AB39256), Phosphorylated Akt (Thermo, Catalog no. 473), HER2 (Roche, Basel, Switzerland, Catalog no. 05278368001), p16 (Roche, Catalog no. 06695221001), Met (Abcam, Catalog no. AB51067), HA-4 (Abcam, Catalog no. AB24480), and 4 microsatellite markers (all from Roche): MLH1 (Catalog no. 06472966001), MSH2 (Catalog no. 05269270001), MSH6 (Catalog no. 5929911001), and PMS2 (Catalog no. 06419216001), RPRM (Sigma, St. Louis, MO Catalog no. SAB1102454), and RPRM-like (Abcam, Catalog no. ab204896). Manual TMA will be prepared as described previously.^[37,38]^ Briefly, paraffin blocks will be obtained and cut and stained by hematoxylin and eosin (H&E) in order to select the best histological area. Subsequently selected tissue area will be placed into the TMA by circling the identified area in the corresponding block. Cylindrical core biopsies will be extracted from each paraffin block using a 20 μm? (please confirm) stylet and placed into a new recipient block. Selected adequate cases had tumors that occupied at least 10% of the core area. Each case will be processed in triplicate to prevent tissue loss during cutting. Sections from each tissue array block will be cut, de-paraffinized and dehydrated for H&E and immunohistochemical procedures.

#### 2.4.8 Gene methylation

Promoter gene methylation on selected coding and noncoding genes that have previously shown promoter regulation by methylation associated with GC will be assessed (Bernal,^[38]^ 18829507 and 19399343). Analysis will be performed by Methylation-Specific PCR and Bisulfite sequencing as described previously^[39]^ using the EZ DNA methylation Gold kit (Zymo Research, Irvine, CA) with minor modifications.

#### 2.4.9 EBV identification

EBV subtypes in patient samples will be assessed using the chromogenic in situ hybridization method with minor modifications.^[25]^

#### 2.4.10 Single nucleotide polymorphism analysis

A significant proportion of GC patients can develop serious toxicity from 5-FU treatment including bone marrow suppression, neuropathy, low white blood cell numbers, fever, infections, nausea, vomiting, severe diarrhea, mouth and digestive tract inflammation, all of which are recorded in the patient history of other cohorts. Subtle personal and population changes in DNA, called SNPs, can account for increases in the risk of 5-FU toxicity; 5-FU metabolism is predominantly hepatic, where the enzyme DPYD is responsible for metabolizing >80% of the drug, producing the inactive metabolite 5,6-dihydroxy-5-FU. It is widely documented that a decreased DPYP activity is associated with severe toxicity.^[31,32,40]^ Nonmetabolized fraction of 5-FU (20%) is transformed by a series of enzymes (e.g., TP, TK), producing the active metabolites that will cause TYMS inhibition, thereby promoting DNA/RNA damage.^[41]^ Variations in TYMS and MTHFR genes (related to reduced folate synthesis, increased 5-FU effect) have been associated with toxicity by treatment with 5-FU. The approach that was used to select the genetic variants consisted of a search in the database, PharmGKB.^[42]^ A total of 6 nonsynonymous SNPs will be analyzed: 4 of them comprise the DPYD gene, 1 for TYMS, and 1 for MTHFR. Analysis will be performed using TaqMan SNP Genotyping Assay technology (Applied Biosystems, Foster City, CA). SNPs will be assessed in DNA isolated from paraffin embedded patient samples.

#### 2.4.11 Sample size and statistical analysis

The minimum sample number will be calculated in order to ensure the goals of the project are fully accomplished. Considering that approximately 90% of GC cases are indeed GAC, at 5% error rate and at 95% confidence interval we originally projected a sample size of 200 patients. However, we have also considered a 15% rate of sample loss (defective samples or patient drop-out), which gives a total of 230 patients to be recruited.

Standard descriptive statistics will be utilized to analyze qualitative and quantitative variables, such as relative and absolute frequencies, frequency tables, average, median, standard deviation, range, and quartiles. A 95% confidence will be considered appropriate for analysis. Descriptive statistics will also be used to characterize the most relevant clinical parameters measured. The association of categorized variables will be performed by chi-squared or Fisher exact test. One arm analysis of variance will compare continuous variables among groups. Survival outcome studies will be accomplished using the Kaplan–Meier method. Prognostic factors will be evaluated according to the Cox proportional hazards regression model.

Principal component analysis of the genes’ variants will be conducted and the association of the first principal components with a small predefined set of genomic alteration signatures will be assessed. To define molecular subgroups, we will utilize unsupervised clustering. The correlation of the molecular subtypes with clinical data (e.g., age, gender, Lauren class) and clinical outcomes (e.g., OS, response rate) will be assessed. Moreover, supervised classification will be performed based on clinical outcomes and the resulting groups of both approaches will be compared with other reported molecular subtypes.

#### 2.4.12 Patient protection/written informed consent forms

All parties guarantee the protection of the patients’ personal records. Patient names are not included in any form in sheet reports, publications, or in any type of publishable document derived from the study with the exception of documents required by law. In cases where nonidentifiable (coded) data transfer is required, CEMP will guarantee the highest confidentiality standards and protection of patients’ personal data. Informed consent forms are elaborated strictly following legal and local regulations. The written informed consent forms, including all changes made throughout the study, must be prospectively approved by the Internal Review Board/Independent Ethics Committee, and CEMP prior to be incorporated into the study.

The investigators, representatives, or healthcare providers will obtain written informed consent forms from every patient or his/her legal representative before any specific activity of the study is performed. Investigators will file and maintain an original copy of all written informed consent forms signed by the patient, an extra original copy will be given to the patient or his/her representative for his/her records.

#### 2.4.13 Monitoring of the study

A registered nurse will monitor this study. The monitor will ensure all procedures are conducted, recorded, and reported in agreement with the standard operating procedures and all applicable regulatory requirements. Since this is a noninterventional study it represents no risk or benefit for the patients.

## 3 Discussion

GAC is a highly heterogeneous disease and the leading cause of death by cancer in Chile, claiming over 3000 deaths every year, and therefore a public health concern. The GCTF study will define the contribution of a subset of genetic, epigenetic, and protein alterations with the clinical outcomes of GAC patients and their response to chemotherapy.

The GCTF study seeks to establish a preventive public health policy based on clinically relevant biomarkers based on personalized medicine. To the best of our knowledge, this study is the first of its kind in Latin America, assessing 120 clinical parameters, and collecting valuable information on the use of antiangiogenic compounds and checkpoint inhibitors on GAC patients. In addition, the study will evaluate EBV prevalence among GAC patients. EBV prevalence in Chilean GC is high at 16% and thus this study protocol may bring to light clinical parameters associated with this infection, along with an updated estimate of its prevalence.^[25]^

The use of 72 predetermined “actionable” targets, profiling a total of 143 known cancer genes (Oncomine Comprehensive assay, Table 1) allows categorization of patients according to their uniquely altered genetic profile. The GCTF strategy could be applied to other countries in the region where GC prevalence is high (see Fig. 1) and may establish the basis for future targeted therapies and a roadmap for future interventional studies that will hopefully improve patient outcomes.

Finally, the GCTF study is a unique example of a coordinated, collaborative effort made by the Chilean Government (CORFO), an academic institution (CITO at Pontifical Catholic University of Chile), and a private initiative CEMP (affiliated to Pfizer Chile) to obtain a comprehensive analysis and a stratification of GAC patients in the Chilean population. It is hoped that lessons learned and recommendations derived from the study will be adopted and incorporated into the clinical practice to make clinical treatment more personalized, cost-effective, and ultimately improve survival outcomes.

## 4 Declarations

### 4.1 Ethics approval and consent to participate

The GCTF is a noninterventional, collaborative, prospective nonconcurrent study that seeks to stratify GAC patients based on their prognosis and therapy response. The study will strictly adhere to all legal requirements, regulations, and general principles established by international agencies governing the ethical conduct in biomedical research on human subjects, following the good clinical practices and the declaration of Helsinki. The GCTF study protocol has been approved by the Ethics Committee of the University hospital (Pontificia Universidad Catolica de Chile, CEC MED UC approval number 16-046, resolution dated April 21, 2016).

### 4.2 Consent for publication

All participants in the study have signed a consent form for publication of data.

### 4.3 Availability of data and material

The datasets used and/or analyzed during the current study are available from the corresponding author on reasonable request.

## Acknowledgments

The authors thank the participating patients of the study, and the University Hospital medical and nursing staff.

## Author contributions

**Conceptualization:** Gareth I Owen, Mauricio P Pinto, Ignacio Retamal, M. Fernanda Fernandez, Betzabe Csiternas, Sebastian Mondaca, Cesar Sanchez, Hector Galindo, Bruno Nervi, Carolina Ibáñez, Francisco Acevedo, Jorge Madrid, Jose Peña, Maria Loreto Bravo, Maria Jose Maturana, Miguel Córdova-Delgado, Nathaly De La Jara, Javiera Torres, Maria Rodríguez-Fernández, Manuel Espinoza, Carlos Balmaceda, Matias Freire, Valentina Garate-Calderón, Fernando Crovari, Paula Jiménez-Fonseca, Alberto Carmona-Bayonas, Ariel Zwenger, Ricardo Armisén, Alejandro H Corvalan, Marcelo Garrido.

**Formal analysis:** Maria Jose Maturana, Miguel Córdova-Delgado, Diego Romero, Nathaly De La Jara, Maria Rodríguez-Fernández, Manuel Espinoza, Carlos Balmaceda, Matias Freire, Valentina Garate-Calderón, Paula Jiménez-Fonseca.

**Funding acquisition:** Gareth I Owen, Ignacio Retamal, Cesar Sanchez, Hector Galindo, Bruno Nervi, Carolina Ibáñez, Francisco Acevedo, Jorge Madrid, Jose Peña, Javiera Torres, Fernando Crovari, Ricardo Armisén, Alejandro H Corvalan, Marcelo Garrido.

**Investigation:** Gareth I Owen, Mauricio P Pinto, Ignacio Retamal, M. Fernanda Fernandez, Betzabe Csiternas, Sebastian Mondaca, Cesar Sanchez, Hector Galindo, Bruno Nervi, Carolina Ibáñez, Francisco Acevedo, Jorge Madrid, Jose Peña, Maria Loreto Bravo, Maria Jose Maturana, Miguel Córdova-Delgado, Diego Romero, Nathaly De La Jara, Javiera Torres, Maria Rodríguez-Fernández, Manuel Espinoza, Carlos Balmaceda, Matias Freire, Valentina Garate-Calderón, Fernando Crovari, Paula Jiménez-Fonseca, Alberto Carmona-Bayonas, Ariel Zwenger, Ricardo Armisén, Alejandro H Corvalan, Marcelo Garrido.

**Methodology:** Gareth I Owen, Mauricio P Pinto, Ignacio Retamal, M. Fernanda Fernandez, Betzabe Csiternas, Sebastian Mondaca, Cesar Sanchez, Hector Galindo, Bruno Nervi, Carolina Ibáñez, Francisco Acevedo, Jorge Madrid, Jose Peña, Maria Loreto Bravo, Maria Jose Maturana, Miguel Córdova-Delgado, Diego Romero, Nathaly De La Jara, Javiera Torres, Maria Rodríguez-Fernández, Manuel Espinoza, Carlos Balmaceda, Matias Freire, Valentina Garate-Calderón, Fernando Crovari, Paula Jiménez-Fonseca, Alberto Carmona-Bayonas, Ariel Zwenger, Ricardo Armisén, Alejandro H Corvalan, Marcelo Garrido.

**Project administration:** Ignacio Retamal, M. Fernanda Fernandez, Betzabe Csiternas, Bruno Nervi, Maria Loreto Bravo, Valentina Garate-Calderón, Ricardo Armisén, Alejandro H Corvalan, Marcelo Garrido.

**Resources:** Ignacio Retamal, Valentina Garate-Calderón.

**Software:** Maria Rodríguez-Fernández, Manuel Espinoza, Carlos Balmaceda.

**Supervision:** Ignacio Retamal, M. Fernanda Fernandez, Betzabe Csiternas, Cesar Sanchez, Hector Galindo, Bruno Nervi, Carolina Ibáñez, Francisco Acevedo, Jorge Madrid, Maria Loreto Bravo, Diego Romero, Ricardo Armisén, Alejandro H Corvalan, Marcelo Garrido.

**Writing – original draft:** Gareth I Owen, Mauricio P Pinto, Carolina Ibáñez, Paula Jiménez-Fonseca, Alberto Carmona-Bayonas, Marcelo Garrido.

**Writing – review & editing:** Gareth I Owen, Mauricio P Pinto, Sebastian Mondaca, Cesar Sanchez, Hector Galindo, Bruno Nervi, Francisco Acevedo, Jorge Madrid, Jose Peña, Maria Loreto Bravo, Maria Jose Maturana, Miguel Córdova-Delgado, Diego Romero, Nathaly De La Jara, Javiera Torres, Maria Rodríguez-Fernández, Manuel Espinoza, Carlos Balmaceda, Matias Freire, Valentina Garate-Calderón, Fernando Crovari, Paula Jiménez-Fonseca, Alberto Carmona-Bayonas, Ariel Zwenger, Ricardo Armisén, Alejandro H Corvalan, Marcelo Garrido.

## Supplementary Material

Supplemental Digital Content
