# Supplementary material for: Chilean Gastric Cancer Task Force: A study protocol to obtain a clinical and molecular classification of a cohort of gastric cancer patients
Source: Medicine (Baltimore). 2018 Apr 20;97(16):e0419. doi: 10.1097/MD.0000000000010419 (PMC5916646; doi:10.1097/MD.0000000000010419)
Supplement: Supplemental Digital Content [file medi-97-e0419-s001.doc]

Supplementary Table S1: General Patient Information.

| **Patient general information** | | **Cancer History** | | **Laboratory Studies** | | **Comorbidity index** | |
| --- | --- | --- | --- | --- | --- | --- | --- |
| variable | Data entered | variable | Data entered | variable | Data entered | variable | Data entered |
| Date of birth | dd/mm/yyyy | Primary tumor site | Distal esophagus, Cardias, body, antrum, pylorus, multiple, fundus, N/A | Basal Bilirubin | <1.5, 1.5-3, 3.1-5, >5, N/A mg/dl | Chronic cardiopathy | Yes or No |
| Gender | M/F | Histological grade | 1, 2, 3, N/A | Basal albumin | >35, 30-35, <30, N/A g/dl | Peripheral vascular disease | Yes or No |
| Weight | In Kg. | Lauren Class | Intestinal, diffuse, mixed, N/A | Basal Alkaline Phosphatase | <85, 85-99, 100-150, >150, N/A U/I | Previous thromboembolic events | Yes or No |
| Waist | In cm. | Signet ring cells | None, <50%, ≥50%, undetermined%, N/A | Basal Lactate Dehydrogenase | ≤250, >250, N/A U/I | Cerebrovascular disease | Yes or No |
| Body mass index (BMI) | Weight (kg)/ Height^2^ (meters) | Plastic linitis | Yes, No, N/A | Hemoglobin | <7, 7-7.9, 8-8.9, 9-9.9, 10-10.9, 11-11.9, 12-12.9, 13-13.9, >14, N/A | Chronic pulmonary disease | Yes or No |
| ECOG | Range 0-4 | Hemorrhagic tumor | No, anemia, hemorrhagic tumor & transfusion, hemorrhagic tumor & history, N/A | Leucocytes | 4,000-11,000, >11,000, N/A per μL | Dementia | Yes or No |
| First diagnosis | dd/mm/yyyy | HER2 overexpression | No (0+, 1+, 2+ & -FISH), Yes (3+), Yes (2+ and +FISH), N/A | Platelets | 100,000-450,000, >450,000, <100,000, N/A per μL | Liver disease | Yes or No |
| TNM | IA/B, IIA, IIB, IIIA, IIIB, IIIC, IV or N/A | Metastatic sites | 1, 2, 3, >3 | CEA | In ng/ml or N/A | Diabetes | Yes or No |
| Surgery on primary tumor | Esophagectomy, partial esopahgectomy & gastrectomy, derivation, laparotomy w/o resection, surgery w/o primary tumor | Measured disease burden | Yes or No |  |  | Chronic renal disease | Yes or No |
| Primary tumor surgery date | dd/mm/yyyy | Lung metastases | No, 1-2, 3-5, >5 |  |  | AIDS | Yes or No |
| Perioperative treatment | None, adjuvant chemo & radio, adjuvant chemo, perioperative chemo, perioperative chemo & radio, other | Peritoneal metastases | No, +nodes on resection, thickened peritoneum, peritoneal mass, permeated omentum fat, intestinal distortion & stenosis |  |  | Other acute complications | Yes or No |
| End of perioperative treatment date | dd/mm/yyyy | Ascites | None, medium, moderate, severe |  |  |  |  |
| Recurrence date | dd/mm/yyyy | Bone metastases | None, 1-2, 3-5, >5 |  |  |  |  |
|  |  | Other metastases | Yes or No (site) |  |  |  |  |
|  |  |  |  |  |  |  |  |

Cancer History, Laboratory Studies, Comorbidity Index. Clinical and laboratory variables registered in Chilean GCTF. Abbreviations used: dd/mm/yyyy: day/month/year, M/F: male/female, w/o: without, ECOG: Eastern Cooperative Oncology Group Performance Status, TNM: Tumor Node Metastasis Classification System, FISH: Fluorescent *in situ* Hybridization, N/A: Not Applicable.

Supplementary Table S2: Chemotherapy regimes used in the GCTF

| **Regime** | **Active drug (s)** | **Options** | **Description (dose, time)** |
| --- | --- | --- | --- |
| DC | Docetaxel, Cisplatin |  | Docetaxel 75 mg/m^2^ +Cisplatin 75mg/m^2^, 3 weeks |
| DCF | Docetaxel, Cisplatin, Fluorouracil | DCF op.1 | 4 weeks, Docetaxel 75 mg/m^2^ day 1+Cisplatin 75 mg/m^2^ day 1 + Fluorouracil 1000 mg/m^2^ continuous for 24 hours on days 1-5 every 4 weeks |
|  |  | DCF op.2 | 3 weeks, Docetaxel 60 mg/m^2^ day 1+Cisplatin 60 mg/m^2^ day 1 + Fluorouracil 750 mg/m^2^ continuous for 24 hours on days 1-4 every 3 weeks |
| DCX | Docetaxel, Cisplatin, Capecitabine |  | Docetaxel 75 mg/m^2^ day 1+Cisplatin 75 mg/m^2^ day 1 +Capecitabine 750 mg/m^2^/12 hours day 1-14 every 3 weeks |
| DOX | Docetaxel, Oxaliplatin, Capecitabine |  | Docetaxel 75 mg/m^2^ day 1+Oxaliplatin 100 mg/m^2^ day 1 +Capecitabine 750 mg/m^2^/12 hours day 1-14 every 3 weeks |
| ECF | Epirubicin, Cisplatin, Fluorouracil |  | Epirubicin 50 mg/m^2^ day 1+Cisplatin 60 mg/m^2^ day 1 + Fluorouracil 200 mg/m^2^ continuous infusion daily every 3 weeks |
| ECX | Epirubicin, Cisplatin, Capecitabine |  | Epirubicin 50 mg/m^2^ day 1+Cisplatin 60 mg/m^2^ day 1 + Capecitabine 750 mg/m^2^/12 hours daily every 3 weeks |
| EOX | Epirubicin, Oxaliplatin, Capecitabine |  | Epirubicin 50 mg/m^2^ day 1+Oxaliplatin 130 mg/m^2^ day 1 + Capecitabine 750 mg/m^2^/12 hours daily every 3 weeks |
| EOF | Epirubicin, Oxaliplatin, Fluorouracil |  | Epirubicin 50 mg/m^2^ day 1+Oxaliplatin 130 mg/m^2^ day 1 + Fluorouracil 200 mg/m^2^ continuous infusion daily every 3 weeks |
| FU + Ox | Fluorouracil Cl or bolus, Oxaliplatin, Leucovorin | FLO | Oxaliplatin 85 mg/m^2^ day 1+Leucovorin 200 mg/m^2^ day 1 + Fluorouracil Cl 2600 mg/m^2^ continuous infusion every 46 hours for 2 weeks |
|  |  | FOLFOX6 | Oxaliplatin 85 mg/m^2^ day 1+Leucovorin 400 mg/m^2^ day 1 + Fluorouracil bolus 400 mg/m^2^ day 1 + Fluorouracil Cl 2400 mg/m^2^ continuous infusion during 46 hours every 2 weeks |
|  |  | Modified FUOX | Oxaliplatin 85 mg/m^2^ day 1+ Fluorouracil Cl 3000 mg/m^2^ continuous infusion during 48 hours every 2 weeks |
| FU + Iri | Fluorouracil Cl or bolus, Irinotecan, Leucovorin | FOLFIRI | Irinotecan 180 mg/m^2^ day 1+ Leucovorin 400 mg/m^2^ day 1 + Fluorouracil bolus 400 mg/m^2^ day 1 + Fluorouracil Cl 2400 mg/m^2^ continuous infusion during 46 hours every 2 weeks |
|  |  | IFL | Irinotecan 80 mg/m^2^ day 1+ Leucovorin 500 mg/m^2^ day 1 + Fluorouracil Cl 2000 mg/m^2^ continuous infusion during 24 hours weekly for 6 weeks, then for 1 week |
| FU + Cispl | Fluorouracil, Cisplatin | FP3w | Cisplatin 75 mg/m^2^ day 1 +Fluorouracil 750 mg/m^2^ continuous infusion during 24 hours daily on days 1-5, every 3 weeks |
|  |  | FP4w | Cisplatin 100 mg/m^2^ day 1 +Fluorouracil 1000 mg/m^2^ continuous infusion during 24 hours daily on days 1-5, every 4 weeks |
| Capecit + Oxalipl | Capecitabine, Oxaliplatin | XELOX | Oxaliplatin 130 mg/m^2^ day 1+Capecitabine 1000 mg/m^2^/12 hours days 1-14 every 3 weeks |
|  |  | Modified XELOX | Oxaliplatin 85 mg/m^2^ day 1+Capecitabine 1000 mg/m^2^/12 hours in days 1-24 every 3 weeks |
| XP | Capecitabine, Cisplatin |  | Cisplatin 85 mg/m^2^ day 1 +Capecitabine 1000 mg/m^2^/12 hours in days 1-24, every 3 weeks |

Therapeutic options for advanced gastric cancer used at the recruiting oncology center. Left hand column is the abbreviation used for each regime. Active drugs, refers to individual chemotherapies in each regime. Final right hand column details strategic dosing and administration timing used at the recruiting oncology clinic

**Supplementary Table S3: Targeted therapies used and registered within the GCTF.**

| **Molecular Target** | **Mechanism of action** | **Targeted agent** | **Dose** |
| --- | --- | --- | --- |
| **HER2** | HER2 monoclonal Antibody | Trastuzumab | 8mg/kg then 6mg/kg |
| **VEGFR** | VEGFR2 monoclonal antibody | Ramucirumab | 80mg/kg |
| **PD-1** | PD-1 monoclonal antibody | Nivolumab | 1mg/2wks |
| **PD-1** | PD-1 monoclonal antibody | Pembrolizumab | 200mg /3wks |
| **CTLA-4** | CTLA-4 monoclonal antibody | Ipilimumab | 3 mg/kg/3wks |

**Supplementary Table S4: Efficacy & Follow up.**

| Variable | Secondary variable | Data entered | Variable | Data entered | Secondary data entered |
| --- | --- | --- | --- | --- | --- |
| Tumor response at 3 months | Target lesions | CR, PR, PD, SD, metastatic disease only not measurable, not measurable | Metastatic disease resection | Resection without residual disease, resection margin microscopically +, resection margin macroscopically + | N/A |
|  | Non-target lesions | N/A, CR, No-CR/No-PD, PD, not measurable | Metastasis surgery date | dd/mm/yyyy | N/A |
|  | New lesions (1 or more) | Yes or No | Palliative radiotherapy? | Yes or No | N/A |
| Tumor response at 6 months | Target lesions | CR, PR, PD, SD, metastatic disease only not measurable, not measurable | Tumor progression after first line chemo? | Yes or No | N/A |
|  | Non-target lesions | N/A, CR, No-CR/No-PD, PD, not measurable | Tumor progression date | dd/mm/yyyy | N/A |
|  | New lesions (1 or more) | Yes or No | Patient receiving second line chemo? | Yes or No | N/A |
| Metastasis surgery | No | No | Second line chemo type | Cisplatin, Docetaxel, Fluoropyrimidine, Irinotecan, Oxaliplatin, Paclitaxel, Other, N/A | N/A |
|  | Yes/ site (s) | Liver, Lungs, lymph node, peritoneal, other | How many lines of chemo? | 1, 2, 3, >3 | N/A |
|  |  |  | Is the patient deceased? | Yes or NO | If Yes, date |

Abbreviations used: CR: Complete Response, PR: Partial Response, PD: Progressive Disease, SD: Stable Disease, dd/mm/yyyy: day/month/year, N/A: Not Applicable.

**Supplementary Table S5: Toxicity according to the Common Toxicity**

**Criteria Adverse Events**

| Variable | Data entered |
| --- | --- |
| Anemia | Grade 0-5 |
| Neutropenia | Grade 0-5 |
| Fever Neutropenia | Grade 3-5 |
| Thrombocytopenia | Grade 0-5 |
| Nausea | Grade 0-5 |
| Vomiting | Grade 0-5 |
| Diarrhea | Grade 0-5 |
| Stomatitis | Grade 0-5 |
| Fatigue | Grade 0-5 |
| Palmar-Plantar Erythrodysesthesia | Grade 0-5 |
| Peripheral neuropathy | Grade 0-5 |
| Alopecia | Grade 0-2 |
| Hyperbilirubineamia | Grade 0-5 |
| Increased aspartate aminotranferase | Grade 0-5 |
| Thromboembolic event | Grade 0-5 |
| Increased Creatinine | Grade 0-5 |
| Heart Failure | Grade 0-5 |
| Hospitalization due to chemotoxicity? | Yes or No |
| Time to reach toxicity grade 3-5 | 0, 1, 2, 3, 4, 5, 6, >6 (months) |
|  |  |

(CTCAE v. 4.1) from NCI registered in the GCTF
